# Molecular Tumor Board-Directed Treatment for Patients with Advanced-Stage Solid Tumors: A Case–Control Real-World Study

**DOI:** 10.3390/curroncol33060304

**Published:** 2026-05-22

**Authors:** Ben Ponvilawan, Dhruv Bansal, Karnav Modi, Beth Gustafson, Lindsey Douglass, Blake Buzard, Marc Roth, Christopher Ward, Timothy Pluard, Janakiraman Subramanian

**Affiliations:** 1 Department of Medicine, Feinberg School of Medicine, Northwestern University, 676 North St. Clair St, Suite 2330, Chicago, IL 60611, USA; 2 Endeavor Health, 2650 Ridge Ave, Evanston, IL 60201, USA; 3 Department of Medicine, University of Missouri-Kansas City, 2411 Holmes St, Kansas City, MO 64108, USA; karnav.modi@umkc.edu; 4 Saint Luke’s Cancer Institute, 4401 Wornall Road, Kansas City, MO 64111, USA; egustafson@saint-lukes.org (B.G.); lnelson5@saint-lukes.org (L.D.); bbuzard@saint-lukes.org (B.B.); mroth@saint-lukes.org (M.R.); christopherward@pittstate.edu (C.W.); tpluard@saint-lukes.org (T.P.); 5 Inova Schar Cancer Institute, 8081 Innovation Park Dr, Fairfax, VA 22031, USA; janakiraman.subramanian@inova.org

**Keywords:** molecular tumor board, targeted therapy, solid tumor, survival outcome, real-world, community oncology

## Abstract

Interpreting and directing treatment based on comprehensive genomic testing for patients with cancer can be challenging. Molecular tumor boards (MTBs) can help by establishing collaborative frameworks to deliver patient care plans with the appropriate incorporation of genomic data. Our retrospective observational study evaluates the impact of MTB on the outcomes of adult patients diagnosed with advanced-stage cancer. Patients between 1 September 2017 and 1 January 2023 were grouped into those who received at least one treatment recommended by the MTB and those who did not. Hazard ratios (HR) for overall survival (OS), progression-free survival (PFS), and time on treatment (ToT) were determined using Kaplan–Meier analysis and multivariate Cox proportional hazards model adjusted for age, stage, line of therapy, and primary site of diagnosis. Of 238 evaluable patients, 138 (58%) received at least one treatment recommended by the MTB. Patient characteristics were well-balanced between the cohorts, except for higher proportions of lung adenocarcinoma, melanoma, and a lower proportion of glioblastoma in the matched cohort. Median OS, PFS, and ToT were all increased in patients on matched treatment compared to those who did not (18.5 months vs. 9.1 months, HR 0.64, 95% confidence interval (CI) 0.43–0.96, *p* = 0.030); 9.7 months vs. 4.3 months, HR 0.64, 95% CI 0.42–0.97, *p* = 0.035; and 4.3 months vs. 2.8 months, HR 0.58, 95% CI 0.41–0.83, *p* = 0.0027, respectively). Our findings show that MTB at a community cancer center is feasible and improves survival among patients with cancer, even after adjusting for confounding variables.

## Simple Summary

Incorporating a multidisciplinary molecular tumor board (MTB) into community cancer care improves overall survival and progression-free survival by providing matching treatment recommendations. Matching medical prescriptions recommended by MTB is achievable, mostly through insurance reimbursement or pharmaceutical company coverage on a compassionate basis, confirming its applicability in real-world settings.

## 1. Introduction

Two decades of technological progress have resulted in significant scientific advances in the science and speed of genomic testing [1]. High-throughput next-generation sequencing (NGS) now enables patient genomic sequencing at a fraction of the historical time and cost [2,3]. In particular, the increased use of NGS in routine oncology practice has enabled the identification of druggable cancer ‘driver’ mutations with a significant impact on patient outcomes [4,5]. The NCI-MATCH (NCT02465060), supported by the National Cancer Institute and the ECOG-ACRIN Cancer Research Group, has shown the importance of identifying actionable mutations that may be matched to a targeted therapy and become potentially effective for cancer therapy [6].

Despite its obvious advantages, the introduction of NGS testing into mainstream oncology practice presents a challenge to the busy practitioner. Knowledge gaps arising from the extensive breadth and depth of ever-evolving NGS methodologies, sequencing capabilities, and frequent updates to curated knowledge bases are beyond the scope of many clinicians’ time and resources [1,2,7]. Precision oncology services, in the form of molecular tumor boards (MTBs), bridge this gap by offering specialized services to earmark patients for approved or experimental targeted therapies. These multidisciplinary tumor boards serve as the central component of precision oncology, being tissue-agnostic and offering expertise in personalized therapy selection. In addition to commercially available targeted therapies, precision oncology services are integral in research through timely referral to genomically stratified clinical trials and the creation of institution-specific NGS patient databases.

With increased accessibility to NGS in clinical practice, multiple cancer centers have adopted the MTBs into their clinical workflow in the past 10 years [8]. An example of this is the Saint Luke’s Center for Precision Oncology (CPO), founded in 2017 to bridge the gap between scientific development and clinical application in an underserved area. The CPO encompasses both research and clinical components, including a molecular tumor board with expertise in bioinformatics, genomics, medical oncology, and molecular pathology. To date, the CPO has registered over 700 patients and discussed over 500 patients in the molecular tumor board.

However, it remains unclear whether incorporating MTB can improve survival outcomes in community-based practice. Previous studies conducted in Italy and Spain showed that patients who received MTB-guided therapy had improved outcomes compared to those who did not, but they were performed in a tertiary, academic cancer center, where resources could potentially be more available [9,10]. Other studies lacked control groups for comparison and had a wide range of responses to MTB-directed therapies (0–67%) [11,12]. Therefore, we conduct a retrospective clinical outcome analysis of patients with advanced solid cancers in a community-based hospital system to evaluate the feasibility and the impact of treatment recommendations by the MTB on survival outcomes.

## 2. Materials and Methods

### 2.1. Study Description

This single-health system, investigator-initiated, retrospective observational study was conducted at the Saint Luke’s Cancer Institute’s (SLCI) Center for Precision Oncology MTB. The protocol was approved through Saint Luke’s Health System Institutional Review Board (IRB code # 18-046, approved 21 March 2022). The board granted a waiver of informed consent.

### 2.2. Participants

Adults of 18 years or older were eligible if molecular testing was completed for a diagnosis of cancer, and the case was reviewed at the SLCI MTB between 1 September 2017 and 1 January 2023, with at least one recommendation made for matched therapy. Matched therapy could include on- or off-label therapies or clinical trials. Patients were excluded if they had no targeted therapy recommendations, continued their previous treatment line, underwent surveillance without any therapy, were lost to follow-up, or were deceased prior to therapy initiation.

A multidisciplinary molecular tumor board, which included experts in bioinformatics, genomics, medical oncology, and molecular pathology, was implemented in 2017 under a master protocol. The MTB performed comprehensive patient reviews to formulate targeted treatment plans based on next-generation sequencing (NGS) panels. Patients were seen in a specialized precision medicine clinic to discuss genomic results and therapy options. Pharmacists assisted with access to targeted on- or off-label therapy and coordinated with clinical trial supervisors for screening in biomarker-directed trials.

### 2.3. Procedures

Data extraction was performed by oncology research staff. Each patient’s electronic medical record (EMR) was reviewed for the collection of patient demographic information, tumor characteristics (e.g., tumor location, histology, staging), cancer-related therapy, NGS reports (including biopsy site, collection date, tumor content, and genomics), and patient outcomes. Any commercially available blood- or tissue-based NGS platform testing was acceptable. MTB recommendations were extracted from the CPO-managed data tracker and confirmed against the EMR note placed after discussions. Eligible NGS panel platforms included Tempus (Chicago, IL, USA), FoundationOne (Boston, MA, USA), Guardant (Palo Alto, CA, USA), Paradigm (Pine Book, NJ, USA), Caris (Irving, TX, USA), and Biocept (San Diego, CA, USA). The genes all of these panels have in common included *AKT1*, *ALK*, *APC*, *AR*, *ARAF*, *ARID1A*, *ATM*, *BRAF*, *BRCA1*, *BRCA2*, *CCND1*, *CCND2*, *CCNE1*, *CDH1*, *CDK4*, *CDK6*, *CDKN2A*, *CTNNB1*, *DDR2*, *EGFR*, *ERBB2*, *ESR1*, *EZH2*, *FBXW7*, *FGFR1*, *FGFR2*, *FGFR3*, *GATA3*, *GNA11*, *GNAQ*, *GNAS*, *HNF1A*, *HRAS*, *IDH1*, *IDH2*, *JAK2*, *JAK3*, *KEAP1*, *KIT*, *KRAS*, *MAP2K1*, *MAP2K2*, *MAPK1*, *MET*, *MLH1*, *MPL*, *MSH2*, *MSH6*, *MTOR*, *MYC*, *MYCN*, *NF1*, *NFE2L2*, *NOTCH1*, *NPM1*, *NRAS*, *NTRK1*, *PALB2*, *PDGFRA*, *PIK3CA*, *PMS2*, *PTEN*, *PTPN11*, *RAF1*, *RB1*, *RET*, *RHEB*, *RHOA*, *RIT1*, *ROS1*, *SMAD4*, *SMO*, *STK11*, *TERT*, *TP53*, *TSC1*, and *VHL*.

If the provider’s subsequent treatment regimen contained any one agent from the tumor board recommendations, then that patient was categorized as matched. If no proposed treatment was included in the patient’s next line of therapy, that patient was categorized as unmatched.

### 2.4. Outcomes

The primary outcomes included OS and ToT, defined as the time from diagnosis to death and the time from the initiation to the discontinuation of therapy, respectively. The secondary endpoint was PFS, defined as the time from diagnosis to radiographic progression interpreted by the treating oncologist.

### 2.5. Statistical Analysis

Demographics variables (age, sex, and race) and tumor characteristics (tumor location, histology, and staging) were reported as raw numbers and percentages. Categorical variables were compared using Fisher’s exact test as a whole group and the mid-*p* exact tests for individual subgroups. Continuous variables were compared using the two-sample *t*-test. Kaplan–Meier plots and Cox proportional hazards multivariate regression model were used to evaluate OS, PFS, and ToT. Multivariate analysis for Cox regression included adjustments for age, stage, line of therapy, and the primary site of the cancer. Of note, age was analyzed as a continuous variable for the multivariate analysis.

## 3. Results

### 3.1. Patient Demographics and Baseline Characteristics

**Figure 1:**
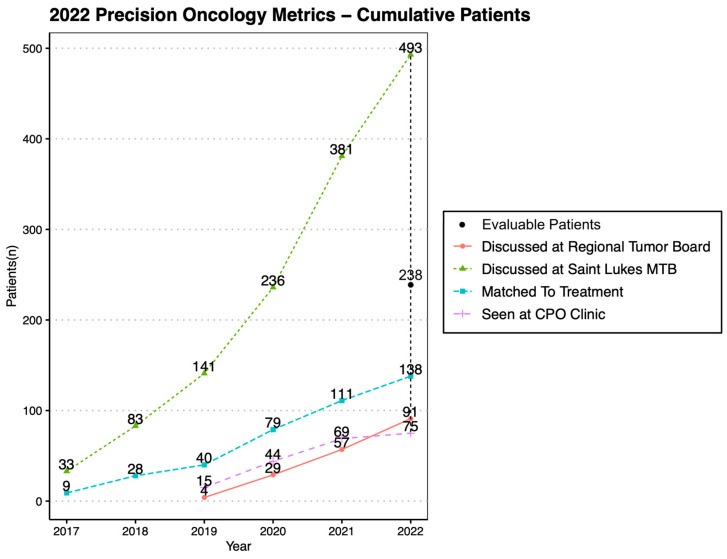
Cumulative number of patients in the SLCI MTB and CPO clinic.

A total of 493 patients were discussed in the MTB at the SLCI between September 2017 and January 2023 (Figure 1). Of all patients being discussed, 238 were evaluable with actionable mutations and started new therapy following the tumor board, with 138 patients (58%) receiving an MTB-matched agent and the rest 100 patients (42%) on an MTB-unmatched regimen (Table 1).

Among the 238 patients, 123 patients (52%) were male. The median patient age was 65.4 years, with 164 patients (69%) falling between the ages of 50–75 and 43 patients (18%) aged >75 years. Caucasians constituted the majority of cases (238 patients, 87%), followed by African Americans (25, 11%), Asians (3, 1%), and Hispanics (2, 1%). The most common primary tumor sites were lung (83, 35%), colon (24, 10%), and breast (22, 9%). The most frequently reported histological finding was lung adenocarcinoma in 59 patients (25%). Most patients had stage IV disease at the time of MTB (160, 67%), while stage I-III disease occurred in 58 (24%) patients. In the study population, 78 patients (33%) were on first-line therapy, 94 patients (39%) on second-line therapy, and 67 patients (28%) on third-line or greater following MTB. Patient characteristics were well balanced between the cohorts, except for a significantly higher proportion of lung adenocarcinoma (33% vs. 14%, *p* = 0.0009) and cutaneous melanoma (9% vs. 2%, *p* = 0.030) in the matched cohort and more glioblastoma in the unmatched cohort (8% vs. 1%, *p* = 0.017).

### 3.2. The Effect of MTB-Matched Therapy on Survival Outcomes and Time-on-Treatment

**Figure 2:**
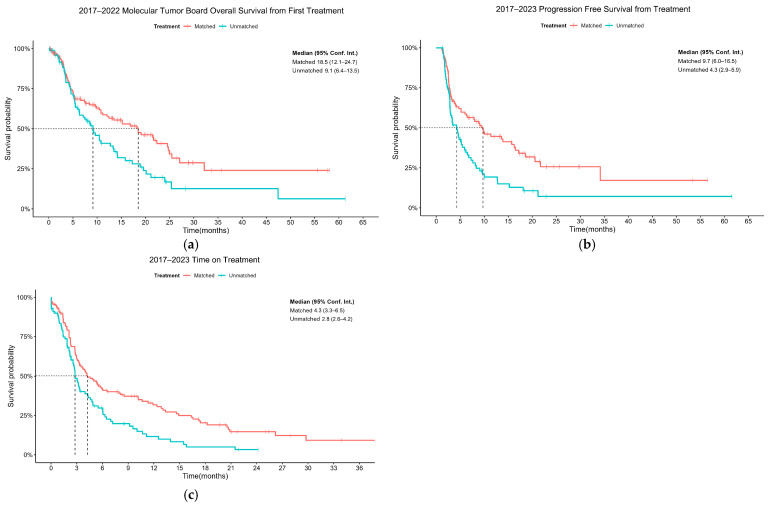
Survival outcomes between MTB-matched vs. MTB-unmatched cohorts by univariate analysis: (**a**) OS, (**b**) PFS, (**c**) ToT. Dashed lines represent median survivals.

The median overall survival (OS) for patients on MTB-matched therapy was 18.5 months (95% confidence interval (CI) 12.1–24.7) versus 9.1 months (95% CI 6.4–13.5) for those who received MTB-unmatched therapy (Figure 2a). The median progression-free survival (PFS) was 9.7 months (95% CI 6.0–16.5) versus 4.3 months (95% CI 2.9–5.9), and median time-on-treatment (ToT) was 4.3 months (95% CI: 3.3–6.5) for MTB-matched versus 2.8 months (95% CI: 2.6–4.2) for MTB-unmatched therapy, respectively (Figure 2b,c). Patients who received MTB-matched therapy had longer OS, PFS, and ToT compared to those who received MTB-unmatched therapy (hazard ratio (HR) for OS 0.61, 95% CI 0.43–0.87, *p* = 0.006; for PFS 0.53, 95% CI 0.37–0.76, *p* < 0.001; and for ToT 0.60, 95% CI 0.44–0.81, *p* < 0.001, respectively). This remained consistent even after adjustments for age, stage, line of therapy, and the primary site of diagnosis (HR for OS 0.64, 95% CI 0.43–0.96, *p* = 0.030; for PFS 0.64, 95% CI 0.42–0.97, *p* = 0.035; and for ToT 0.58, 95% CI 0.41–0.83, *p* = 0.0027) (Table 2).

### 3.3. Clinical Factors Associated with Survival Outcomes

Age, cancer stage at diagnosis, and line of therapy (other lines vs. 2nd line) were not associated with differential survival outcomes in the multivariate analysis. Compared to primary colorectal cancer, primary lung, breast, gastro-esophageal, pancreatic, and gynecologic cancers did not have different survival outcomes. The primary site of the tumor also did not differentially affect the duration of cancer therapy. Of note, those with hepatobiliary and unknown primary were independently associated with worsened PFS, but not OS, while primary eye and otolaryngologic tumors had poorer OS and PFS than colorectal cancer. (Table 2).

### 3.4. Means of Therapy Acquisition and Financial Feasibility of MTB-Recommended Therapy

**Figure 3:**
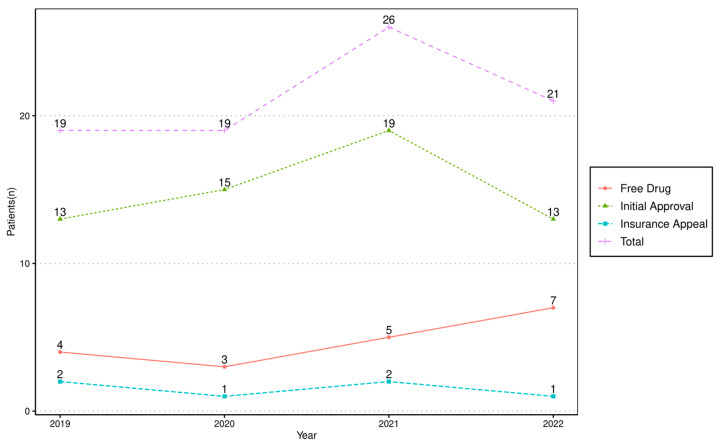
Means of medication acquisition of MTB-recommended therapy.

**Figure 4:**
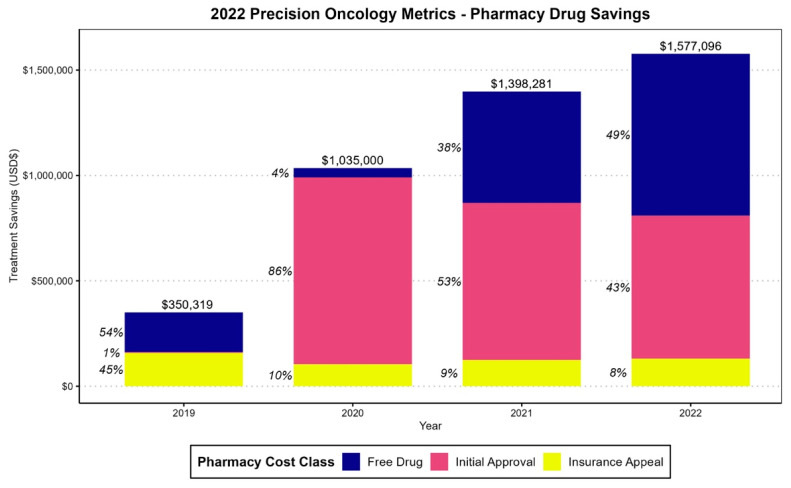
Annual pharmacy drug savings breakdown by financial source.

To further evaluate whether the treatment recommendations by MTB were financially feasible within the current system, we investigated the means of medication acquisition for the MTB-recommended therapy and the amount of medication cost covered by each means. Among the 85 out of 98 cases that had evaluable financial data from 2019 to 2022, the initial medication requests covered 70.6% of cases, and insurance appeals covered further 7.1% of cases (Figure 3). In total, the MTB obtained $4.3 million worth of free medication supplies from the drug manufacturer recommended for patients through MTB-matched therapy, while successfully overturning insurance denials through MTB appeals totaling $515,000 from 2019 to 2022 (Figure 4).

## 4. Discussion

Our real-world study revealed that patients who received matched treatments achieved a 40% improvement in OS and PFS compared to those who received unmatched treatments in a pan-cancer focused MTB, even after multiple corrections for age, stage, line of therapy, and the primary site of diagnosis. This nearly twofold improvement in OS and PFS aligns with and broadens the results from previous studies, which have reported the positive impact of precision oncology approaches on survival outcomes in specific cancer subtypes such as lung and colorectal cancer [6,13]. This supports the role of the MTB and the need for its access globally. In addition, ToT was similarly longer in the matched cohort, suggesting that MTB-guided therapies not only delay disease progression but also allow patients to remain on treatment longer, likely improving quality of life and reducing the need for frequent therapy changes. These improvements are consistent with studies demonstrating that tailoring treatment based on molecular profiling leads to better disease control and similar or fewer toxicities compared to empiric approaches [14].

Our center successfully increased access to targeted therapies through the multidisciplinary nature of our MTB, which allows pharmacists and social workers to pursue insurance overrides and foundation funding for MTB recommendations [15,16,17]. However, despite having 58% of all patients seen in MTB matched with targeted therapies based on actionable mutations, which is already higher than that reported in other precision medicine studies, they were significantly enriched in those with lung adenocarcinoma [4,18]. This likely reflects the high prevalence of actionable molecular alterations, such as those with *EGFR* and *ALK*, in lung adenocarcinoma, which have well-established targeted therapies [19]. Conversely, the lower match rates in glioblastoma and other tumor types may highlight the need for continued research into effective targeted therapies for tumors with fewer actionable alterations. This imbalance underscores the current limitations of precision oncology, where certain cancers disproportionately benefit from advances in molecular targeting.

The racial representation of patients presented in MTB may also serve in the improvement of the results. While most patients were Caucasian (87%), the inclusion of minority populations, albeit small, is important to ensure the generalizability of findings. Prior studies have highlighted disparities in access to genomic testing and precision oncology treatments among minority groups, and efforts to improve diversity in genomic databases and ensure equitable access to precision oncology services remain crucial [20].

There are several limitations of our study that warrant careful interpretation of the results. First, the study had a small sample size and was retrospective in nature, limiting the capability to adjust the analysis based on specific genomic alterations. Especially, the number of patients in some disease sites, such as pancreatic, brain, gastro-esophageal, gynecologic, and eye/otolaryngologic cancers, is small and can restrict the generalizability of the study results to these particular types of cancer. Second, some patient population imbalances may occur in the analysis. Patients with lung adenocarcinoma and melanoma were more prevalent in the matched treatment cohort, while those with glioblastoma were enriched in the unmatched treatment cohort. Patients in the matched cohort were more likely to receive first-line therapy instead of later-line. These may potentially introduce bias and be mitigated by the implementation of multivariate Cox regression analysis; nonetheless, unmeasured confounders may still exist. Third, performance status data were not evaluable in this study; however, only patients who received a new line of therapy after MTB were included. Fourth, patients who received off-label therapies, despite matched therapies to their cancer’s genetic aberrations, may have an uncertain treatment response and duration compared to approved therapies, which can jeopardize the overall efficacy of matched therapies. Lastly, a convenience sample was used to select patients for the analysis due to staffing constraints. This is to help mitigate unintended selection bias, as all patients presenting at MTB during the study period were eligible for this study.

## 5. Conclusions

Our study presents convincing evidence that incorporating a multidisciplinary MTB to provide matching treatment recommendations improves overall survival and progression-free survival, with most prescriptions being either reimbursable by the insurance or covered by pharmaceutical companies on a compassionate basis. This supports further establishment of MTB in oncology practice to improve patient outcomes.

## Figures and Tables

**Table 1:** Baseline characteristics of the patients in this study.

	Total		Matched		Unmatched			*p*
	No.	(%)	No.	(%)	No.	(%)		
Total	238	(100%)	138	(58%)	100	(42%)		
Sex							1.00	
Male	123	(52%)	71	(51%)	52	(52%)		0.934
Female	115	(48%)	67	(49%)	48	(48%)		0.934
Race							0.477	
Caucasian	208	(87%)	121	(88%)	87	(87%)		0.873
African American	25	(11%)	15	(11%)	10	(10%)		0.829
Asian	3	(1%)	2	(1%)	1	(1%)		0.812
Hispanic	2	(1%)	0	(0%)	2	(2%)		<0.450
Age							0.143	
Median age (years)	65.4		66.4		64.3			
20–49	31	(13%)	20	(14%)	11	(11%)		0.440
50–75	164	(69%)	88	(64%)	76	(76%)		0.976
>75	43	(18%)	30	(22%)	13	(13%)		0.085
Stage							0.248	
I	9	(4%)	7	(5%)	2	(2%)		0.243
II	14	(6%)	8	(6%)	6	(6%)		0.940
III	35	(15%)	19	(14%)	16	(16%)		0.634
IV	160	(67%)	94	(68%)	66	(66%)		0.732
Unstaged	2	(1%)	2	(1%)	0	(0%)		0.806
Stage Not Applicable	13	(5%)	4	(3%)	9	(9%)		0.050
Unknown	5	(2%)	4	(3%)	1	(1%)		0.364
Line of Therapy							0.014	
1st	78	(33%)	53	(38%)	25	(25%)		0.030
2nd	94	(39%)	56	(41%)	38	(38%)		0.691
≥3rd	66	(28%)	29	(21%)	37	(37%)		0.007
Histology							5 × 10^−4^	
Lung Adenocarcinoma	59	(25%)	45	(33%)	14	(14%)		0.0009
Colorectal Adenocarcinoma	24	(10%)	10	(7%)	14	(14%)		0.097
Breast Ductal Carcinoma	17	(7%)	6	(4%)	11	(11%)		0.058
Melanoma	14	(6%)	12	(9%)	2	(2%)		0.030
Lung Squamous Cell Carcinoma	14	(6%)	7	(5%)	7	(7%)		0.544
Prostatic Adenocarcinoma	11	(5%)	5	(4%)	6	(6%)		0.409
Glioblastoma	10	(4%)	2	(1%)	8	(8%)		0.017
Esophagogastric Adenocarcinoma	10	(4%)	5	(4%)	5	(5%)		0.614
Pancreatic Adenocarcinoma	8	(3%)	2	(1%)	6	(6%)		0.071
Cholangiocarcinoma	7	(3%)	6	(4%)	1	(1%)		0.151
Non-cutaneous Squamous Cell Carcinoma, All Other Primaries	7	(3%)	6	(4%)	1	(1%)		0.151
Lung Small Cell Carcinoma	6	(3%)	2	(1%)	4	(4%)		0.254
Endometrial Carcinoma	4	(2%)	3	(2%)	1	(1%)		0.551
Sarcoma	4	(2%)	1	(1%)	3	(3%)		0.231
Urothelial Carcinoma	3	(1%)	1	(1%)	2	(2%)		0.382
Breast Lobular Carcinoma	3	(1%)	2	(1%)	1	(1%)		0.812
Appendiceal Adenocarcinoma	3	(1%)	2	(1%)	1	(1%)		0.812
Other Histologies	34	(14%)	21	(15%)	13	(13%)		0.640

**Table 2:** Multivariate Cox analysis for survival outcomes, adjusted by age, stage, line of therapy, and the primary site of diagnosis.

	Reference	N	Group	OS				PFS				ToT			
				HR	CI		*p*	HR	CI		*p*	HR	CI		*p*
Matched Treatment	Unmatched	138	Matched	0.64	(0.43–0.96)		0.030	0.64	(0.42–0.97)		0.035	0.58	(0.41–0.83)		0.003
Age				1.00	(0.99–1.02)		0.711	0.99	(0.98–1.00)		0.192	1.00	(0.98–1.01)		0.57
Stage	IV	9	I	0.37	(0.11–1.20)		0.097	0.66	(0.23–1.86)		0.432	0.73	(0.33–1.61)		0.44
		14	II	1.18	(0.49–2.84)		0.713	1.50	(0.65–3.46)		0.342	1.31	(0.59–2.93)		0.51
		35	III	1.06	(0.61–1.84)		0.850	1.39	(0.78–2.47)		0.263	1.57	(0.99–2.47)		0.05
		2	Unstaged	1.80	(0.72–4.50)		0.210	1.52	(0.59–3.94)		0.385	0.91	(0.41–2.00)		0.81
		13	Staging Not Applicable	0.68	(0.08–6.09)		0.727	NE	NE		NE	1.08	(0.22–5.44)		0.92
		5	Unknown	0.30	(0.04–2.36)		0.251	0.52	(0.11–2.48)		0.409	0.57	(0.13–2.54)		0.46
Line of Therapy	2nd Line	78	1st Line	1.06	(0.67–1.67)		0.798	0.74	(0.44–1.24)		0.252	0.81	(0.54–1.21)		0.30
		66	≥3rd	1.26	(0.80–1.99)		0.326	1.70	(1.07–2.71)		0.025	1.00	(0.68–1.49)		0.99
Primary site	Colon	83	Lung	1.46	(0.73–2.90)		0.282	1.61	(0.80–3.24)		0.186	1.31	(0.73–2.33)		0.37
		22	Breast	0.97	(0.43–2.23)		0.951	1.34	(0.60–3.00)		0.480	0.99	(0.50–1.98)		0.99
		17	Skin	0.76	(0.27–2.19)		0.616	0.96	(0.33–2.76)		0.939	1.14	(0.52–2.49)		0.74
		15	Genitourinary	0.78	(0.29–2.11)		0.622	1.26	(0.48–3.34)		0.640	1.22	(0.56–2.68)		0.62
		13	Brain *	1.55	(0.76–3.19)		0.228	1.43	(0.67–3.06)		0.359	1.05	(0.55–1.99)		0.89
		12	Hepatobiliary	2.00	(0.79–5.07)		0.146	3.37	(1.31–8.66)		0.012	1.44	(0.64–3.26)		0.38
		10	Gastric and Esophageal	1.26	(0.40–3.93)		0.692	1.24	(0.43–3.58)		0.695	0.76	(0.28–2.10)		0.60
		9	Gynecological	1.42	(0.56–3.61)		0.456	1.53	(0.56–4.19)		0.407	1.03	(0.45–2.35)		0.94
		8	Pancreas	2.17	(0.84–5.62)		0.109	2.00	(0.66–6.03)		0.219	1.58	(0.65–3.87)		0.32
		6	Eyes and Otolaryngologic	4.06	(1.04–15.85)		0.044	3.86	(1.12–13.28)		0.032	1.52	(0.42–5.45)		0.52
		5	Tumor of Unknown Origin	0.76	(0.17–3.50)		0.729	3.95	(1.07–14.67)		0.040	2.40	(0.86–6.71)		0.10
		14	Other	1.29	(0.42–3.96)		0.657	1.68	(0.52–5.48)		0.389	1.01	(0.40–2.53)		0.99

* Unadjusted hazard ratio reported due to limited statistical power. Abbreviation: NE, not evaluable.

## Data Availability

The data presented in this study are available upon request from the corresponding author due to the presence of individual patient data.

## Abbreviations

The following abbreviations are used in this manuscript:

NGS	Next-generation sequencing
MTB	Molecular tumor board
CPO	Center for Precision Oncology
SLCI	Saint Luke’s Cancer Institute
OS	Overall survival
PFS	Progression-free survival
ToT	Time-on-treatment
HR	Hazard ratio
CI	Confidence interval
NE	Not evaluable
EGFR	Epidermal growth factor receptor
ALK	Anaplastic lymphoma kinase
